# Supplementary material for: Volatile Compound Abundance Correlations Provide a New Insight into Odor Balances in Sauce-Aroma Baijiu
Source: Foods. 2022 Dec 5;11(23):3916. doi: 10.3390/foods11233916 (PMC9739518; doi:10.3390/foods11233916)
Supplement: Supplementary file 1 [file foods-11-03916-s001.zip › Supplementary Table S1.pdf]

Supplementary Table S1. Chemical standards and their quantification parameters.

Compounds	RI_{Cal} [a]	RI_{Lit} [b]	LOD (ug/L)	LOQ (ug/L)	Recovery (%)	R^2	IS [f]
Acetaldehyde	692	689[e]	11	36.7	97	0.996	IS1
1-Propanal	801	784[e]	12.5	41.7	96	0.991	IS1
Ethyl formate	814	813[d]	128.9	429.7	98	0.996	IS3
1,1-Diethoxymethane	829	826[d]	76.1	253.7	101	0.992	IS1
Acetone	835	813[e]	43	143.3	93	0.993	IS1
Isobutyraldehyde	855	855[e]	54.2	180.7	101	0.992	IS1
1,1-Diethoxyethane	878	871[d]	284	946.7	94	0.99	IS1
2-Butanone	892	894[e]	18.9	63.0	96	0.996	IS1
Ethyl acetate	896	876[d]	45.4	151.3	101	0.996	IS3
Methanol	904	910[e]	97.2	324.0	99	0.998	IS1
2-Methylbutyraldehyde	916	916[e]	24.9	83.0	93	0.996	IS1
Ethyl propanoate	928	928[c]	21.9	73.0	100	0.998	IS3
3-Methylbutyraldehyde	935	932[e]	44.5	148.3	92	0.996	IS1
Ethyl isobutyrate	960	960[e]	44.9	149.7	98	0.994	IS3
Propyl acetate	973	977[e]	54.2	180.7	100	0.997	IS3
2-Pentanone	996	996[e]	32.1	107.0	89	0.991	IS1
Ethyl butyrate	1021	1023[c]	37.4	124.7	101	0.994	IS3
2-Butanol	1027	1016[d]	32.4	108.0	93	0.999	IS1
1-Propanol	1035	1032[d]	18.1	60.3	87	0.991	IS1
1,1-Diethoxy-3-methylbutane	1068	1068[c]	21.6	72.0	92	0.995	IS3
Ethyl isovalerate	1071	1072[e]	329	1096.7	103	0.995	IS1
1,1-Diethoxy-2-methylbutane	1079	1079[e]	69.2	230.7	98	0.992	IS1
Isobutanol	1092	1093[e]	60.4	201.3	101	0.992	IS1
2-Pentanol	1112	1118[d]	13	43.3	104	0.991	IS1
Isoamyl acetate	1122	1126[e]	65.9	219.7	99	0.998	IS3
Ethyl valerate	1132	1139[e]	51	170.0	100	0.998	IS3
1-Butanol	1139	1139[c]	17.4	58.0	91	0.998	IS1
2-Methyl butanol	1204	1203[c]	8.8	29.3	87	0.993	IS3
3-Methyl butanol	1209	1209[c]	21.6	72.0	102	0.996	IS1
Ethyl hexanoate	1239	1239[c]	3.81	12.7	103	0.999	IS3
1-Pentanol	1255	1255[e]	74.2	247.3	100	0.992	IS1
3-Hydroxy-2-butanone	1291	1292[e]	231.1	770.3	95	0.993	IS3
Hexanol	1350	1353[e]	26.8	89.3	94	0.998	IS1
Ethyl heptanoate	1357	1357[c]	26.5	88.3	106	0.999	IS3
Ethyl lactate	1359	1359[c]	67.1	223.7	93	0.993	IS3
Butyl caproate	1427	1427[e]	7.7	25.7	90	0.995	IS3
Acetic acid	1435	1438[c]	73	243.3	97	0.998	IS2

Ethyl octanoate	1440	1439 [c]	13.2	44.0	104	0.998	IS2
Furfural	1471	1441 [d]	86.3	287.7	100	0.999	IS2
Benzaldehyde	1502	1502 [d]	27.3	91.0	91	0.993	IS2
Propionic acid	1528	1530 [c]	145	483.3	98	0.999	IS2
Ethyl nonanoate	1531	1530 [e]	12.5	41.7	108	0.998	IS2
Meso-2,3-butanediol	1536	1538 [e]	792	2640.0	86	0.996	IS2
(2R,3R)-(-)-2,3-Butanediol	1545	1545 [e]	179.2	597.3	94	0.991	IS2
Isobutyric acid	1562	1564 [e]	16.3	54.3	104	0.993	IS2
Hexyl hexanoate	1609	1609 [c]	9.92	33.1	98	0.998	IS2
Butyric acid	1615	1614 [c]	28.4	94.7	77	0.992	IS2
Ethyl decanoate	1644	1648 [e]	48.5	161.7	92	0.995	IS2
Isovaleric acid	1652	1647 [e]	11.6	38.7	105	0.998	IS2
Furfuryl alcohol	1665	1665 [e]	89.6	298.7	92	0.993	IS2
Valeric acid	1725	1720 [e]	33.8	112.7	99	0.992	IS2
Ethyl phenylacetate	1782	1782 [c]	5.17	17.2	103	0.991	IS2
Ethyl laurate	1841	1840 [c]	32.5	108.3	105	0.997	IS2
Hexanoic acid	1849	1844 [c]	26.3	87.7	76	0.998	IS2
Benzyl alcohol	1868	1866 [e]	5.9	19.7	86	0.992	IS2
Ethyl 3-phenylpropanoate	1884	1884 [c]	12.8	42.7	101	0.994	IS2
2-Phenylethanol	1920	1899 [d]	18	60.0	92	0.994	IS2
Heptanoic acid	1953	1953 [e]	37.9	126.3	95	0.993	IS2
Ethyl myristate	2049	2070 [e]	35.3	117.7	103	0.992	IS2
Octanoic acid	2055	2055 [c]	475	1583.3	89	0.997	IS2
Nonanoic acid	2162	2162 [c]	208	693.3	97	0.998	IS2
Ethyl hexadecenoate	2251	2251 [c]	116	386.7	103	0.999	IS2
Ethyl palmitate	2258	2251 [c]	58.1	193.7	102	0.993	IS2
Ethyl oleate	2479	2479 [c]	222	740.0	94	0.996	IS2
Ethyl linoleate	2525	2530 [e]	56.8	189.3	76	0.991	IS2

[a] RI_{Cal}, linear retention index calculated.

[b] RI_{Lit}, Reference linear retention index from literature.

[c] Zhao, D., Shi, D., Sun, J., Li, A., Sun, B., Zhao, M., ... & Zheng, F. (2018). Characterization of key aroma compounds in Gujinggong Chinese Baijiu by gas chromatography–olfactometry, quantitative measurements, and sensory evaluation. *Food Research International*, *105*, 616-627.

[d] Pino, J. A., Tolle, S., Gök, R., & Winterhalter, P. (2012). Characterisation of odour-active compounds in aged rum. *Food chemistry*, *132*(3), 1436-1441.

[e] Standard Reference Data Act. (2021), *NIST Chemistry WebBook, SRD 69*, Retrieved from https://webbook.nist.gov/chemistry/

[f] Corresponding internal standard (IS) of aroma compounds. IS1: Amyl acetate; IS2: 2-methyl hexanoic acid; IS3: tertiary amyl alcohol.
