# Supplementary figures and images for: Volatile Compound Abundance Correlations Provide a New Insight into Odor Balances in Sauce-Aroma Baijiu
Source: Foods. 2022 Dec 5;11(23):3916. doi: 10.3390/foods11233916 (PMC9739518; doi:10.3390/foods11233916)

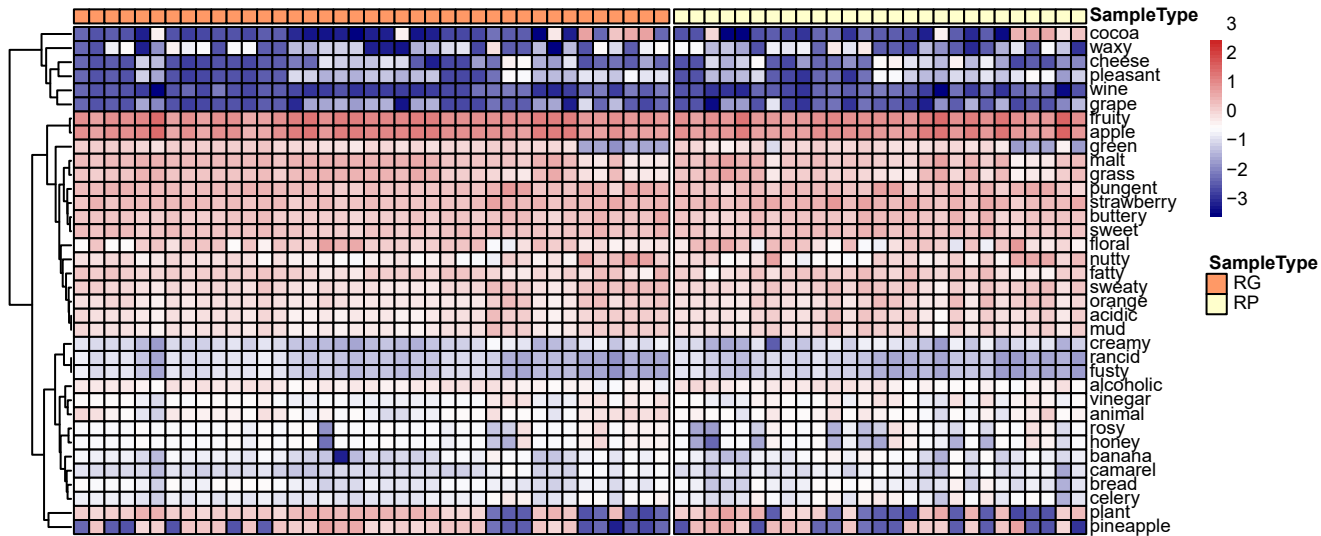

Supplement: Supplementary file 1 [file foods-11-03916-s001.zip › Supplementary Figure S1.pdf]

**A**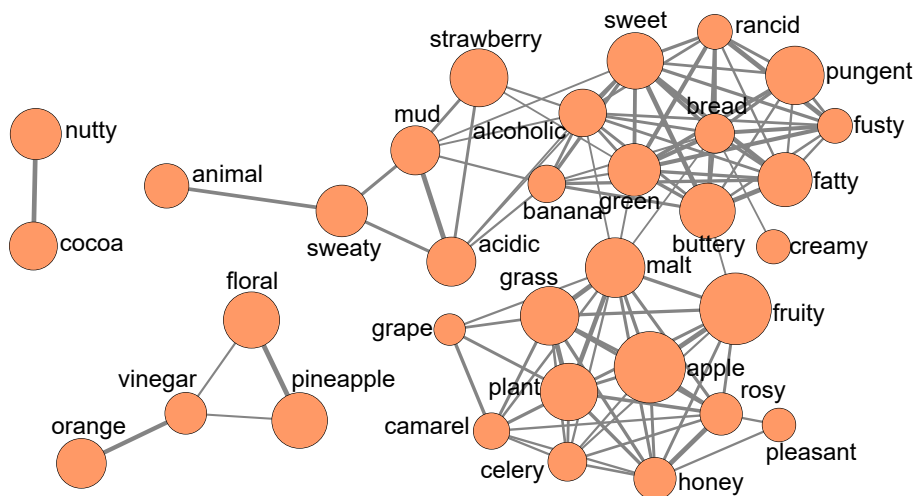**B**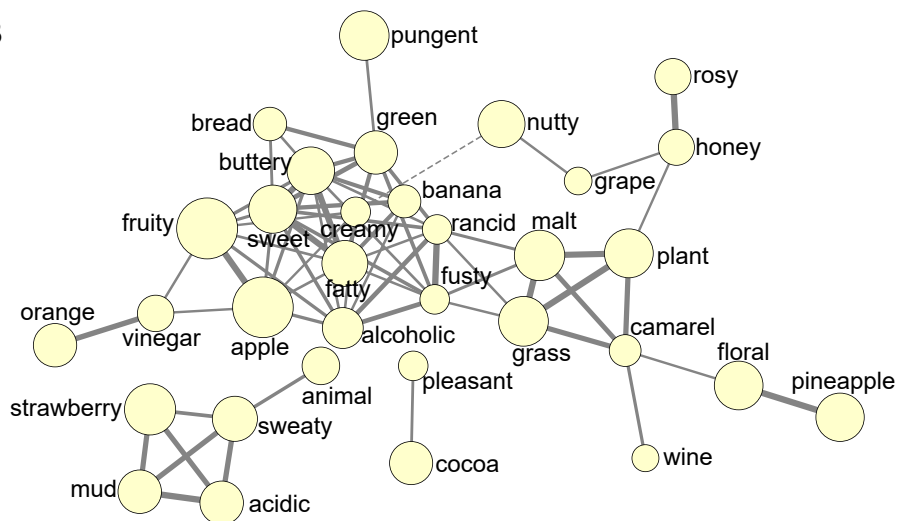

Supplement: Supplementary file 1 [file foods-11-03916-s001.zip › Supplementary Figure S2.pdf]

**A**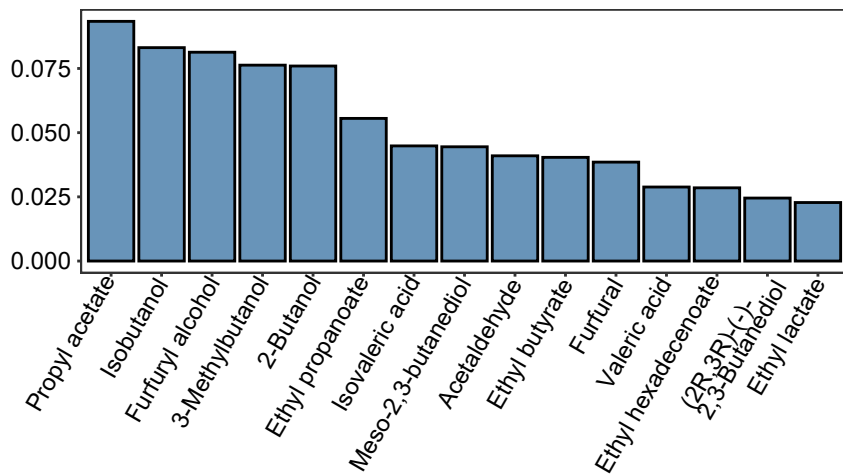**B**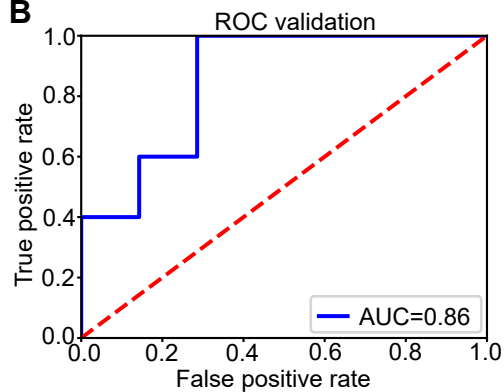**C**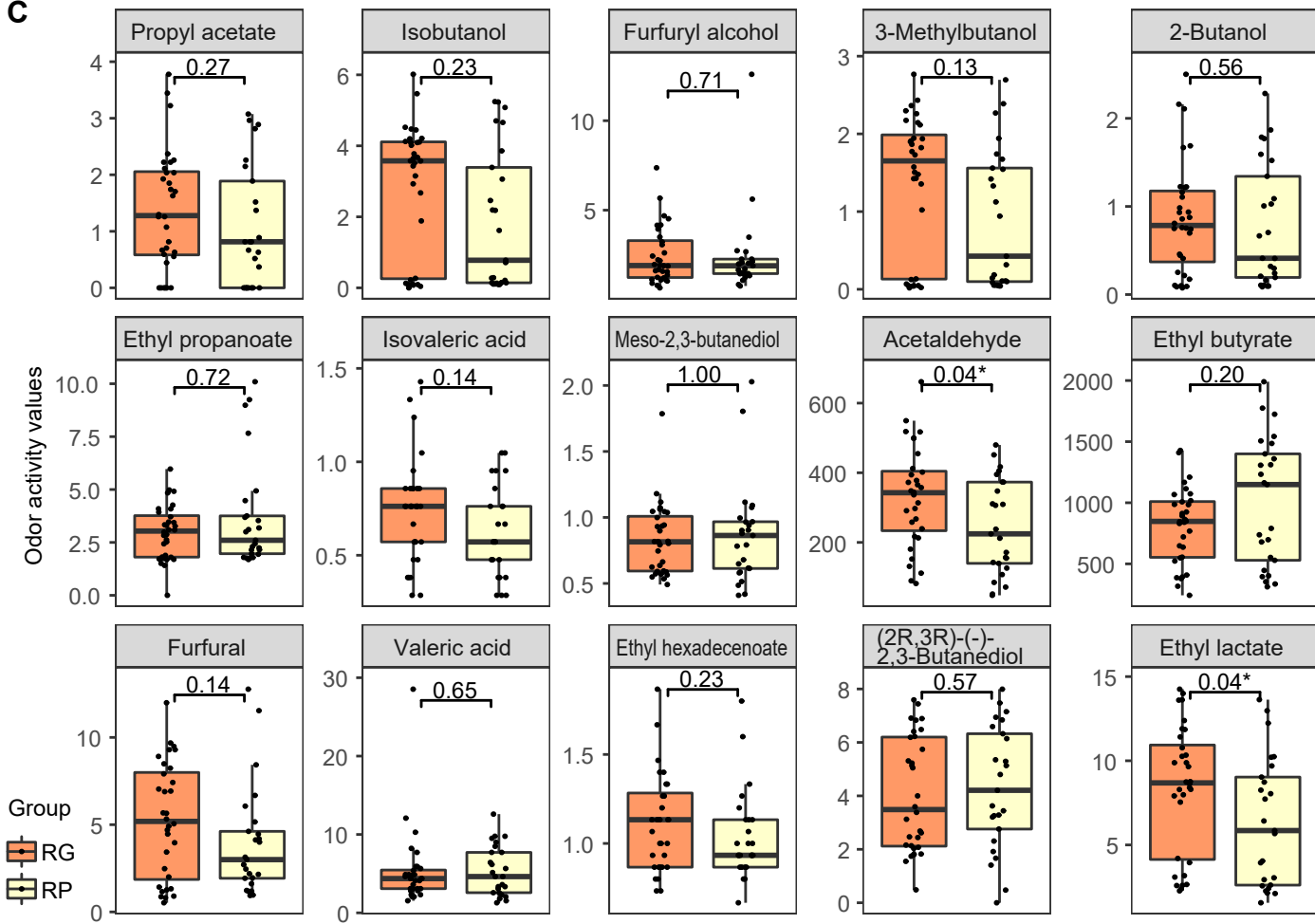

Supplement: Supplementary file 1 [file foods-11-03916-s001.zip › Supplementary Figure S3.pdf]

**A**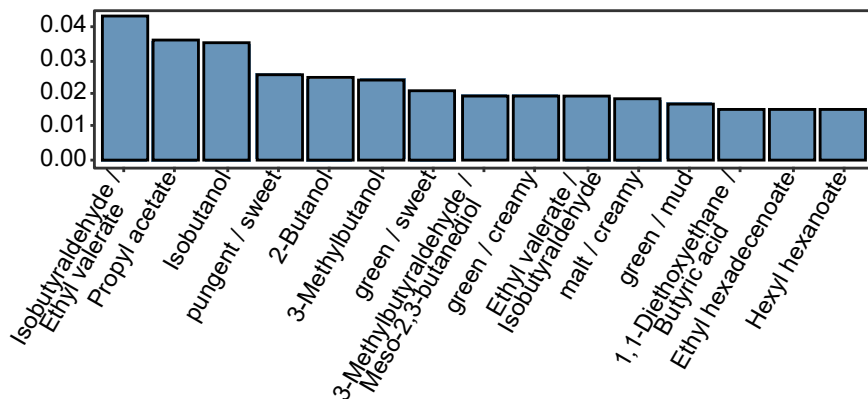**B**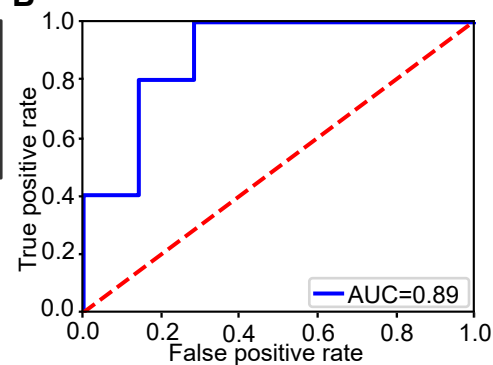**C**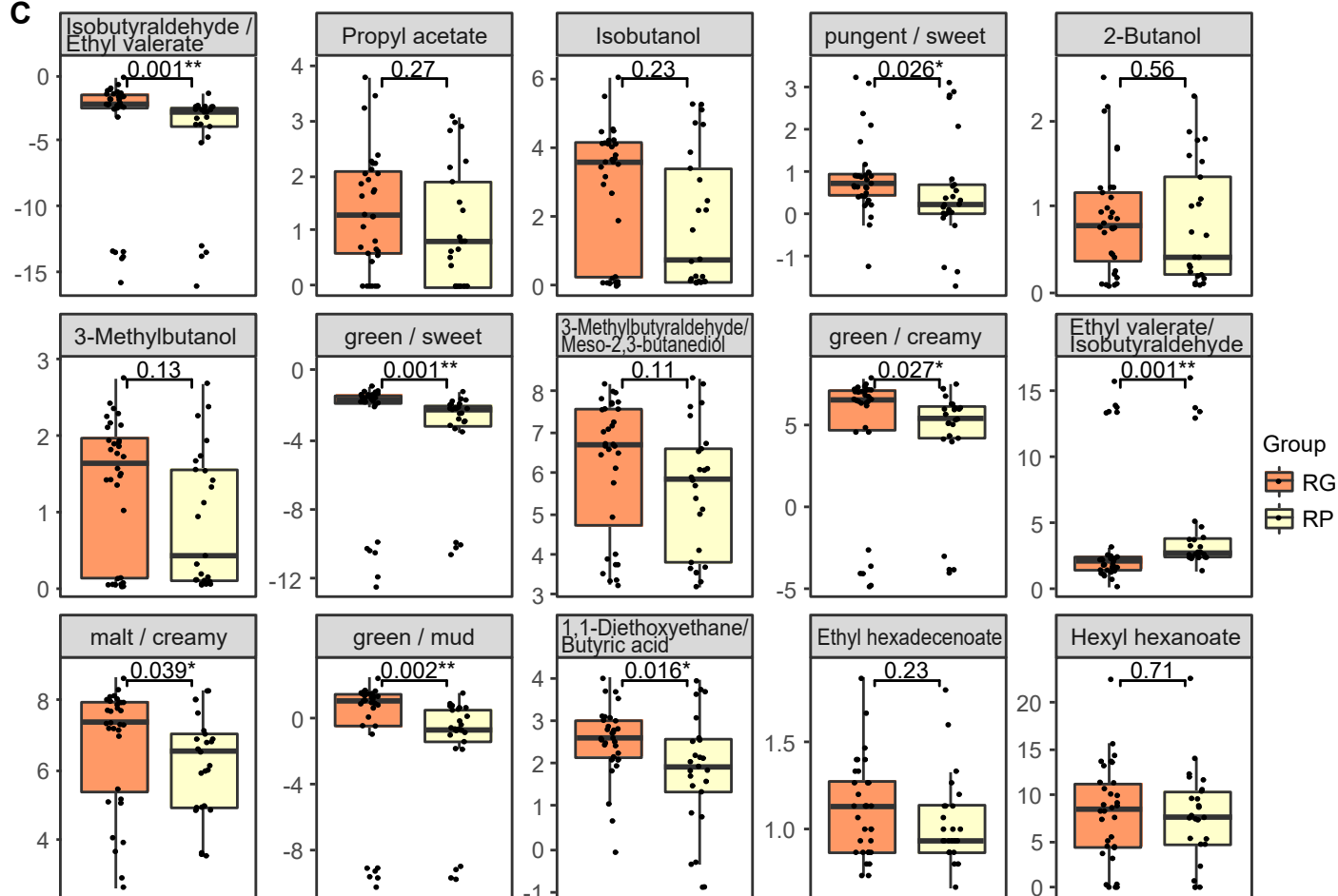

Supplement: Supplementary file 1 [file foods-11-03916-s001.zip › Supplementary Figure S4.pdf]

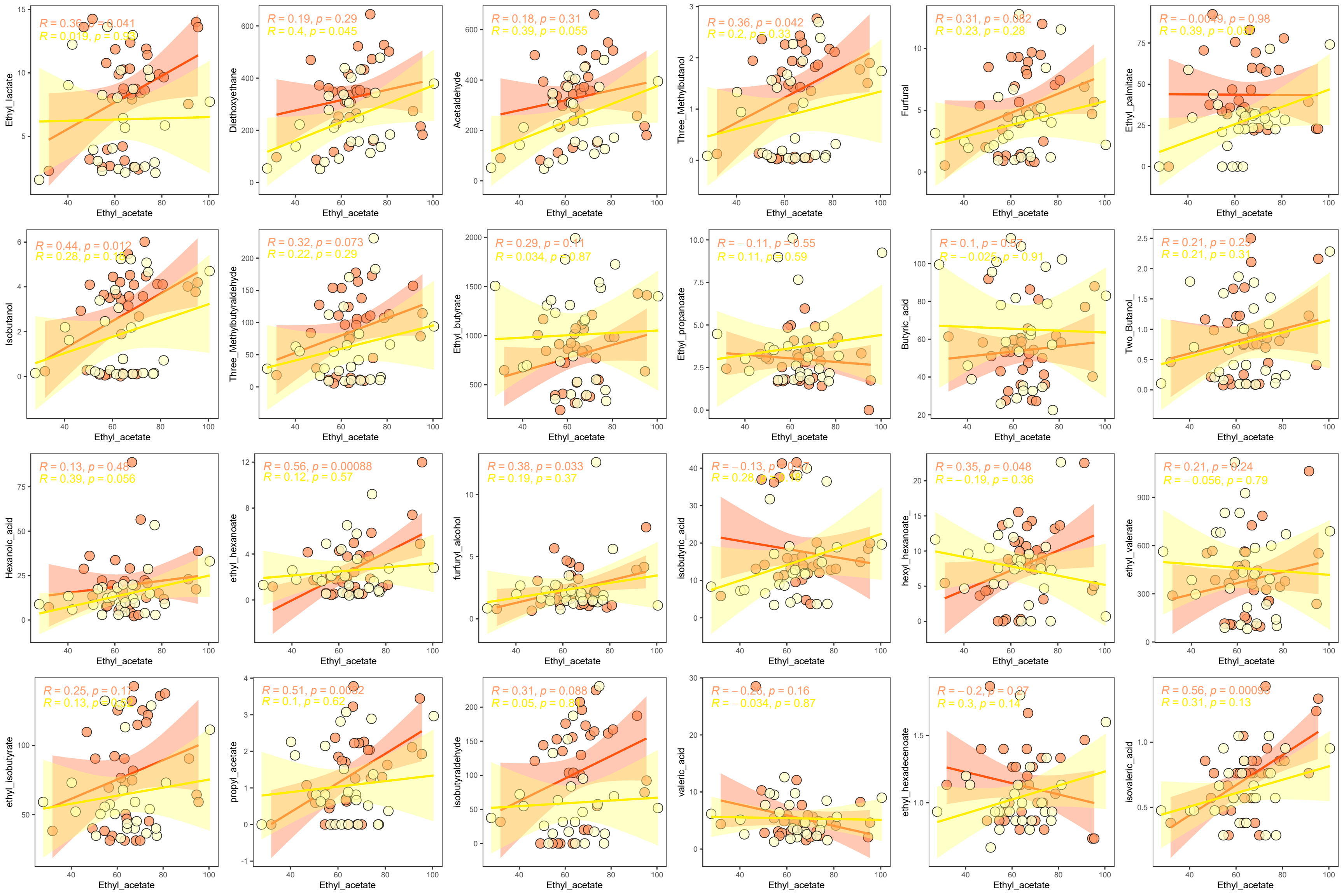

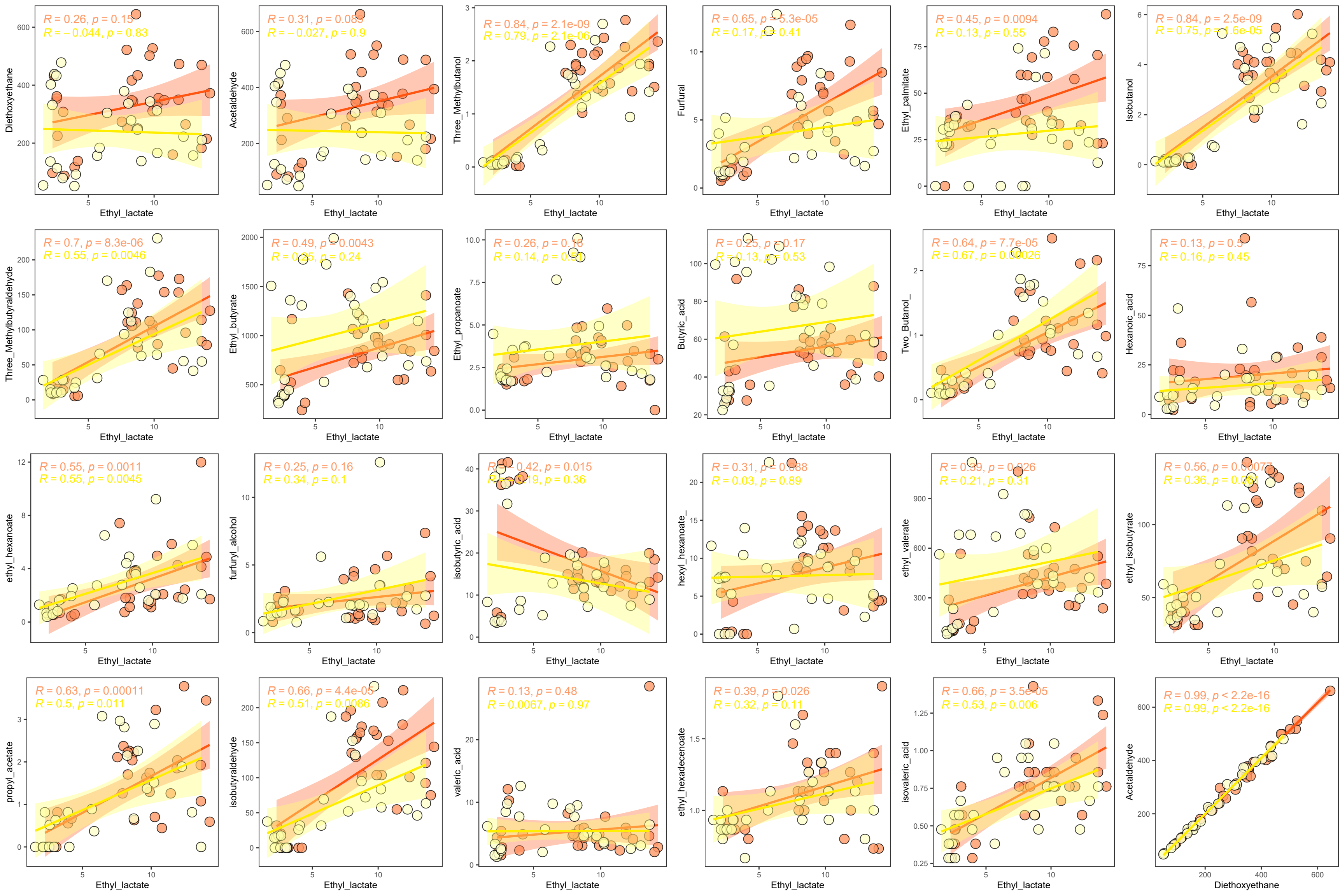

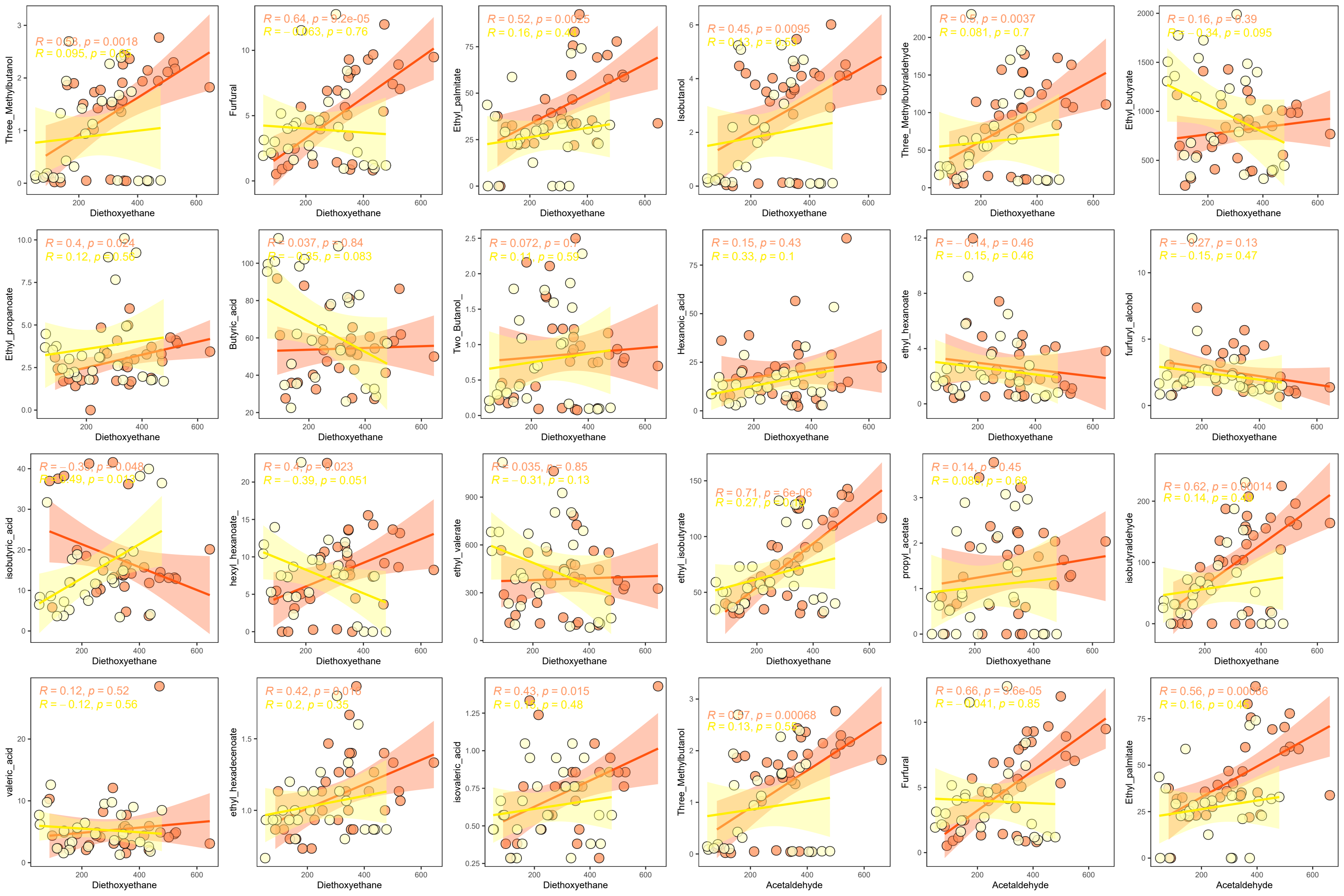

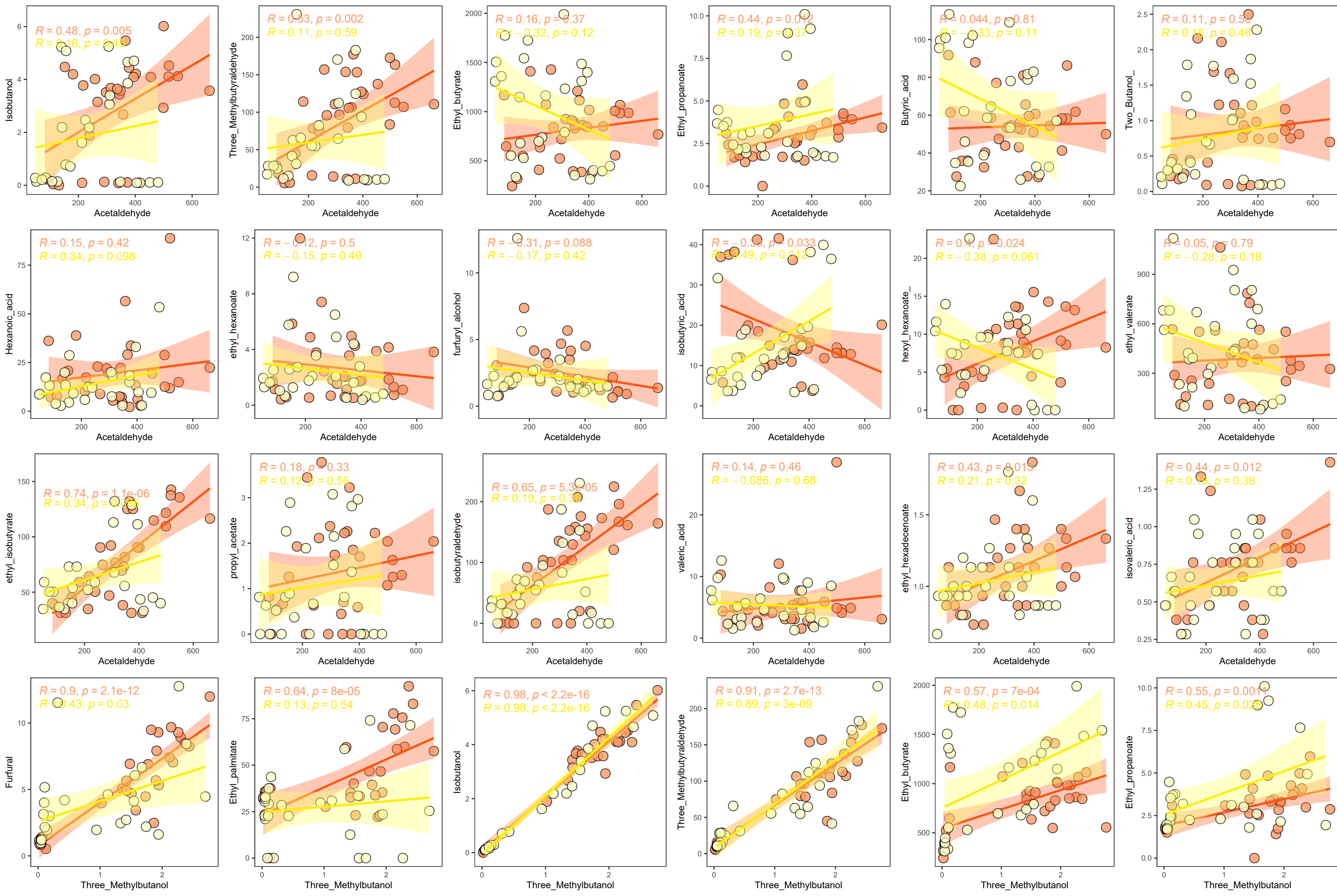

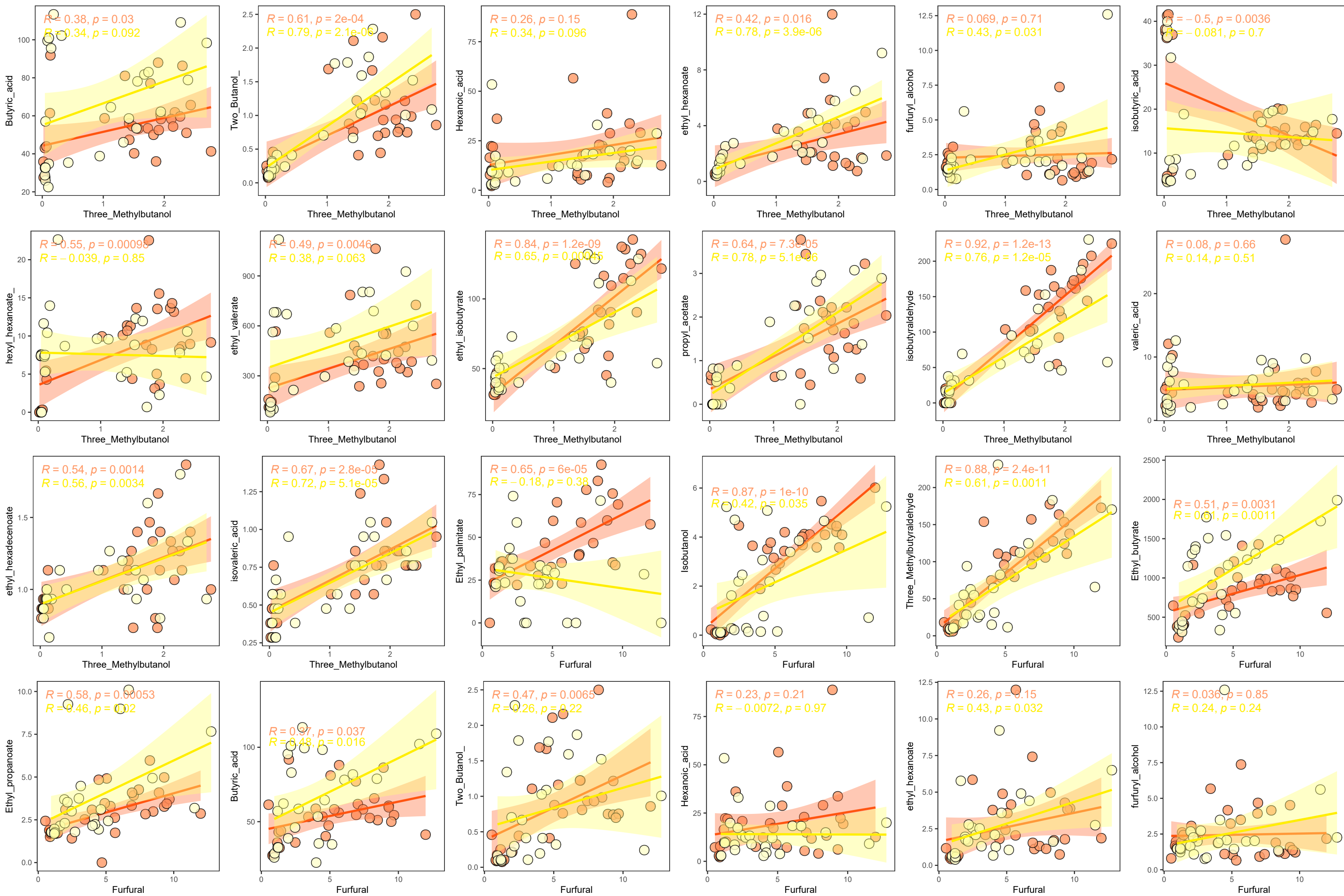

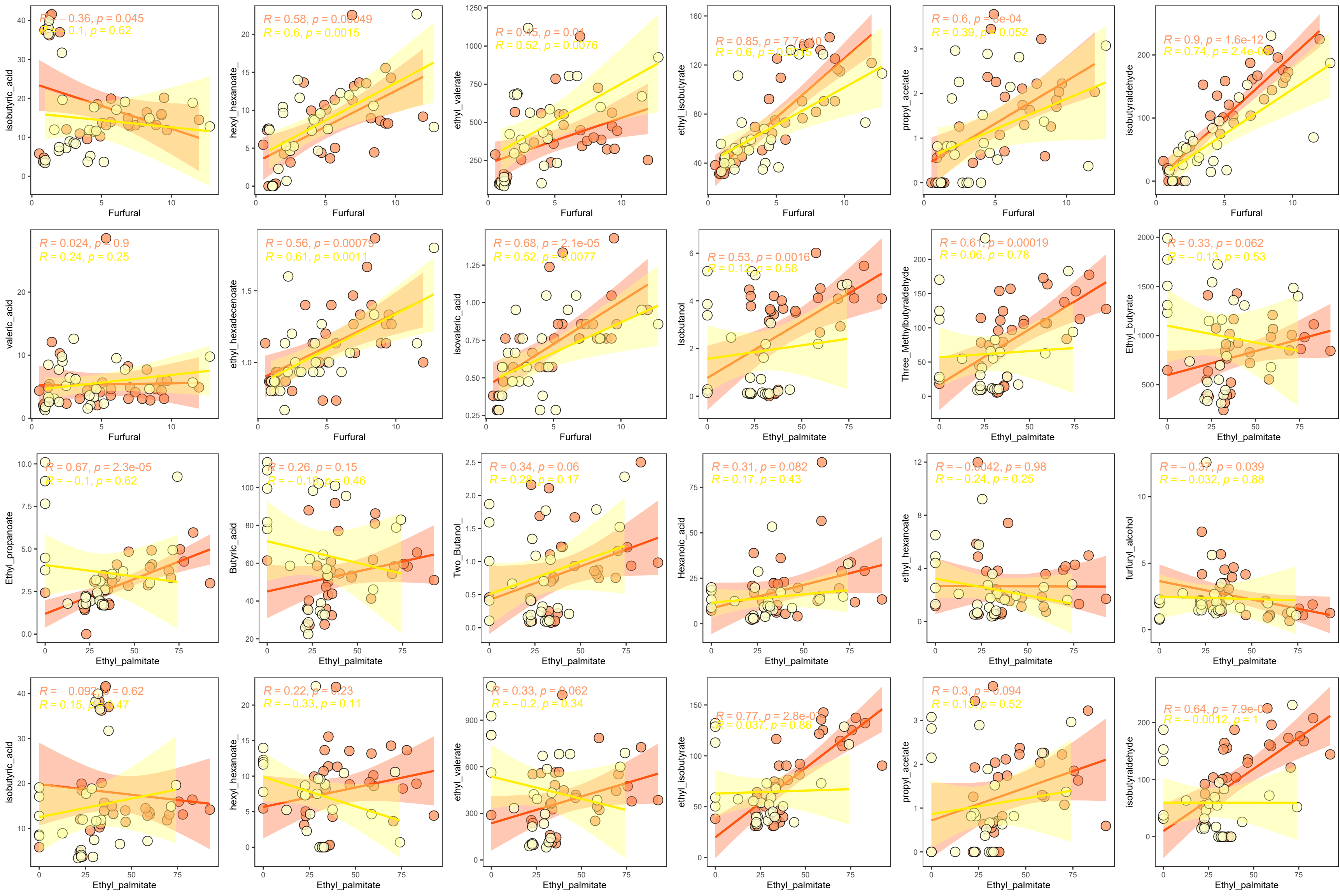

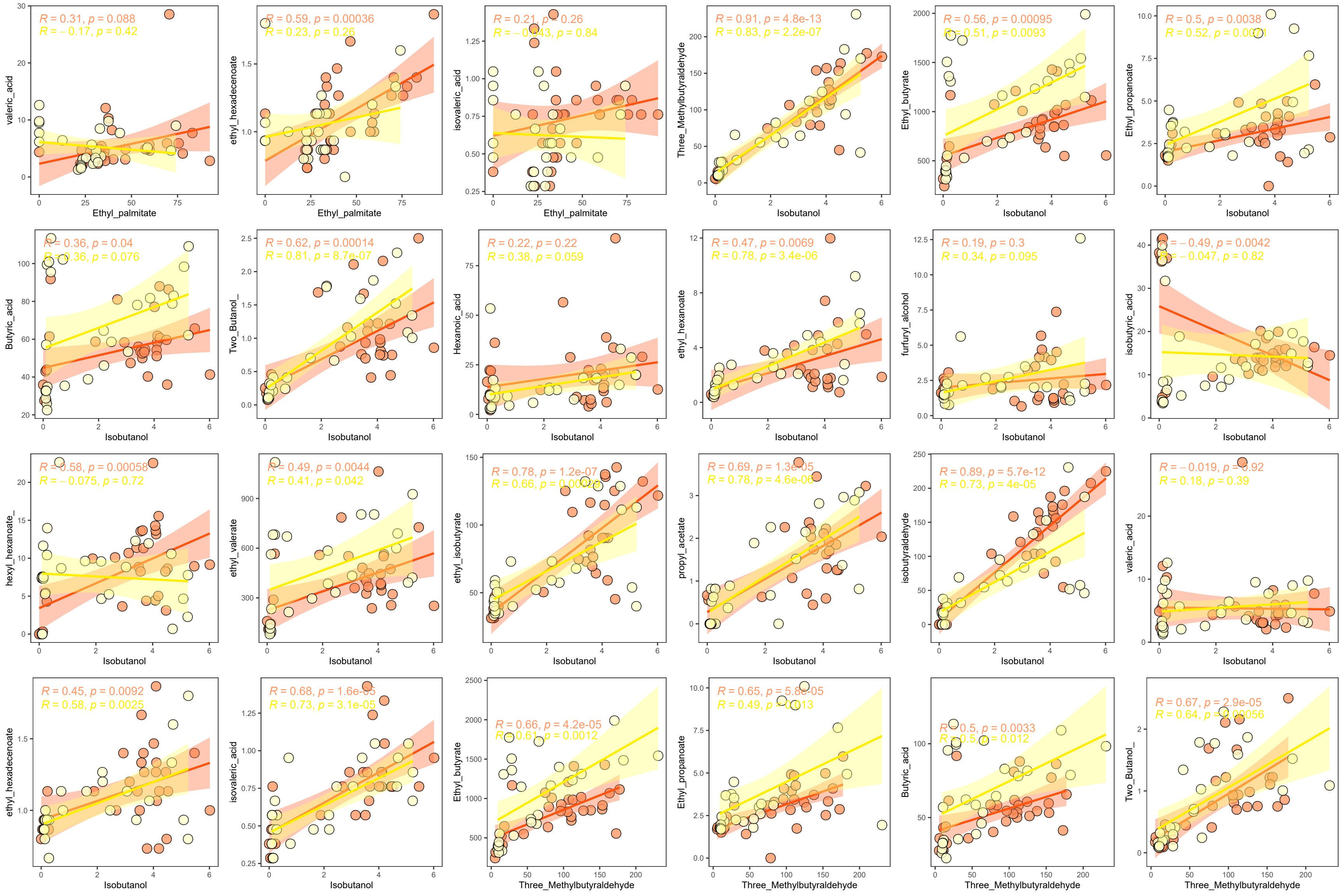

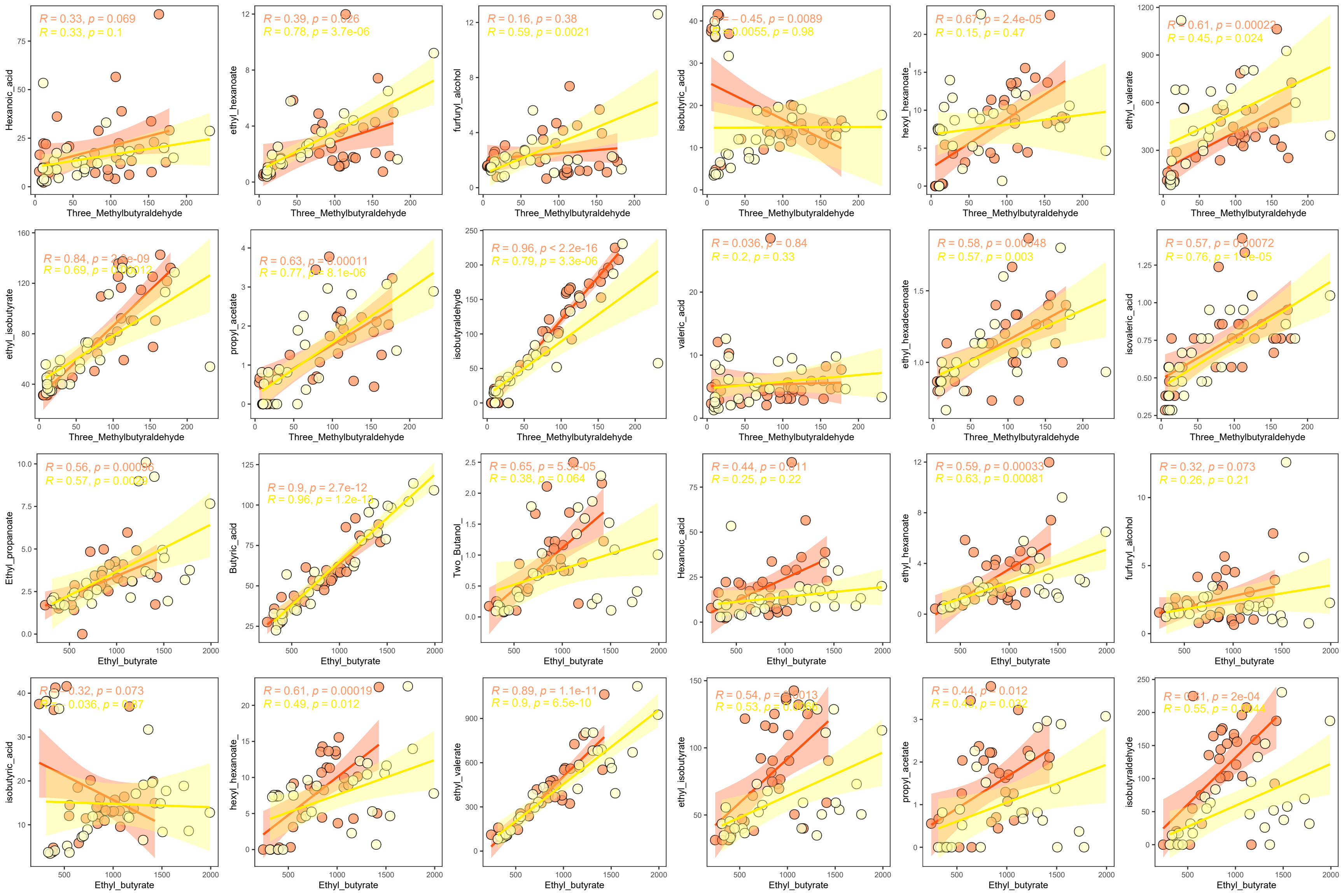

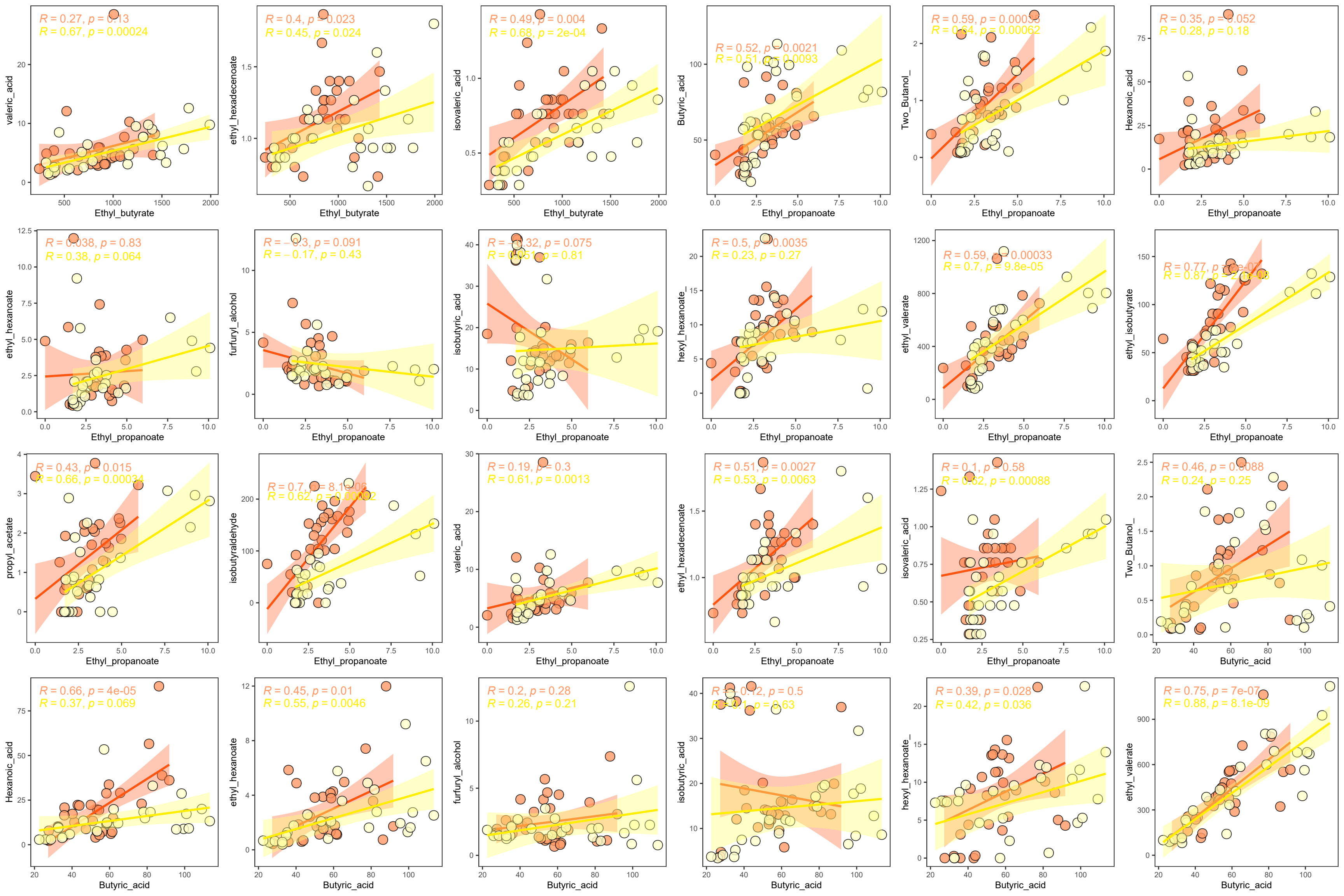

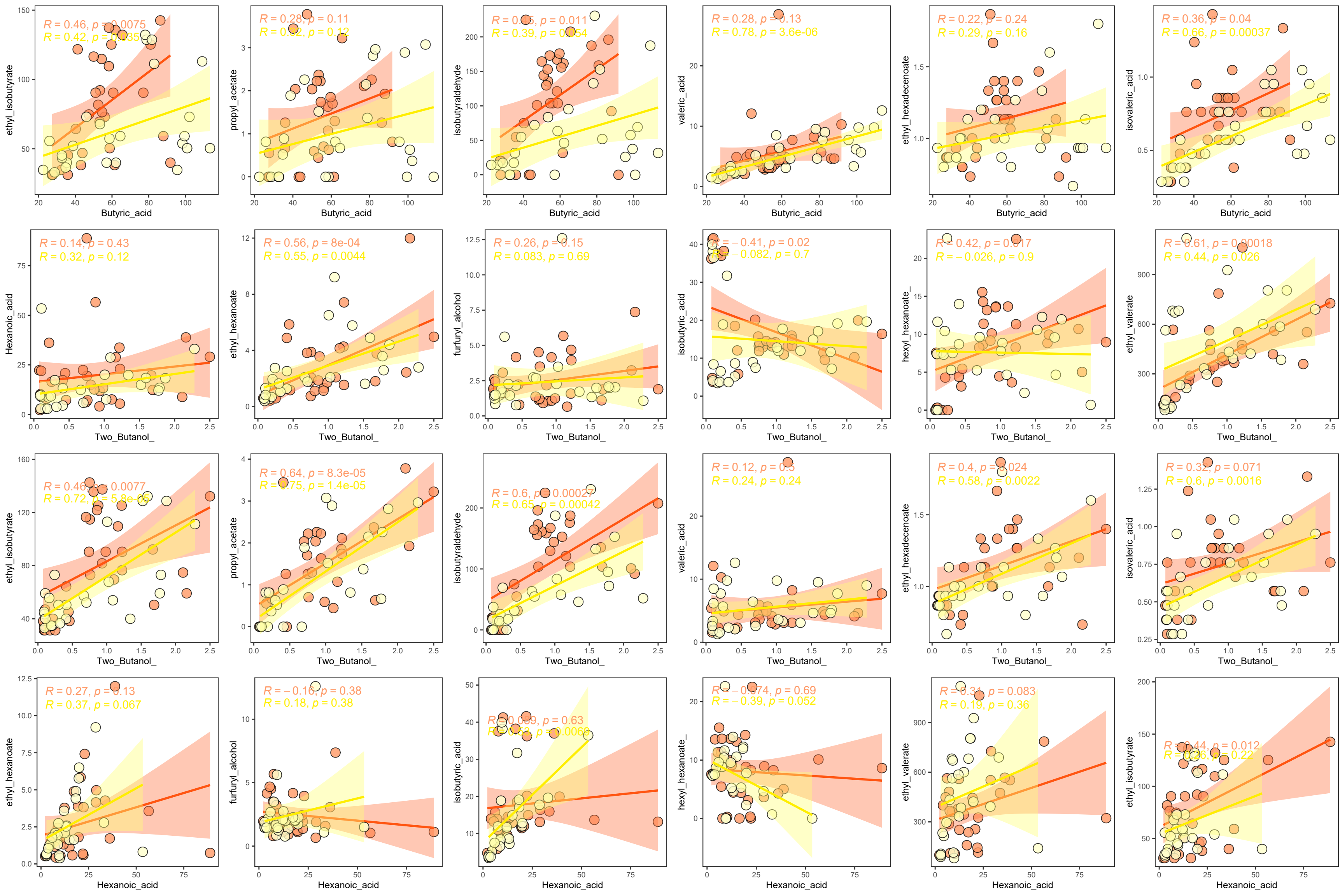

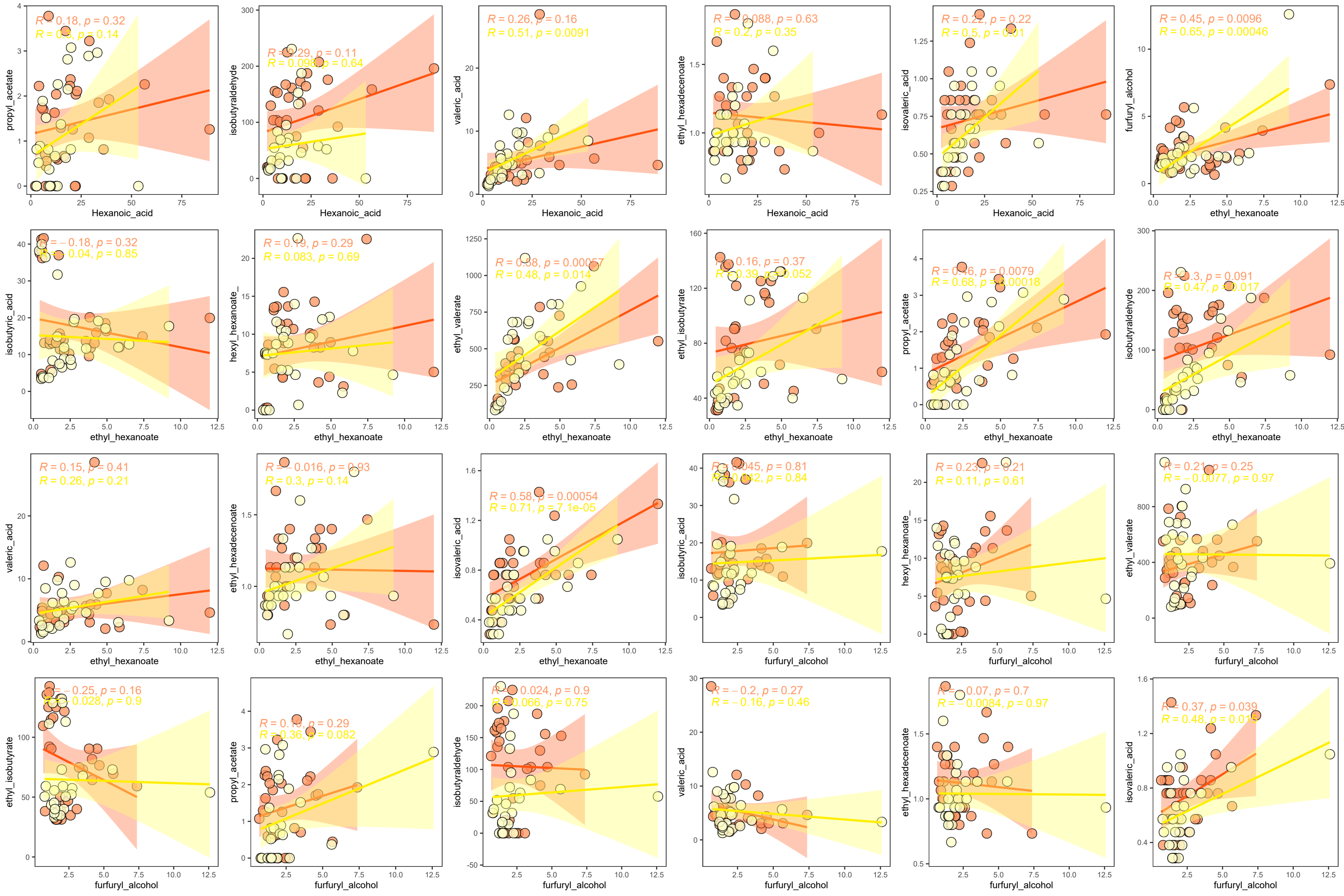

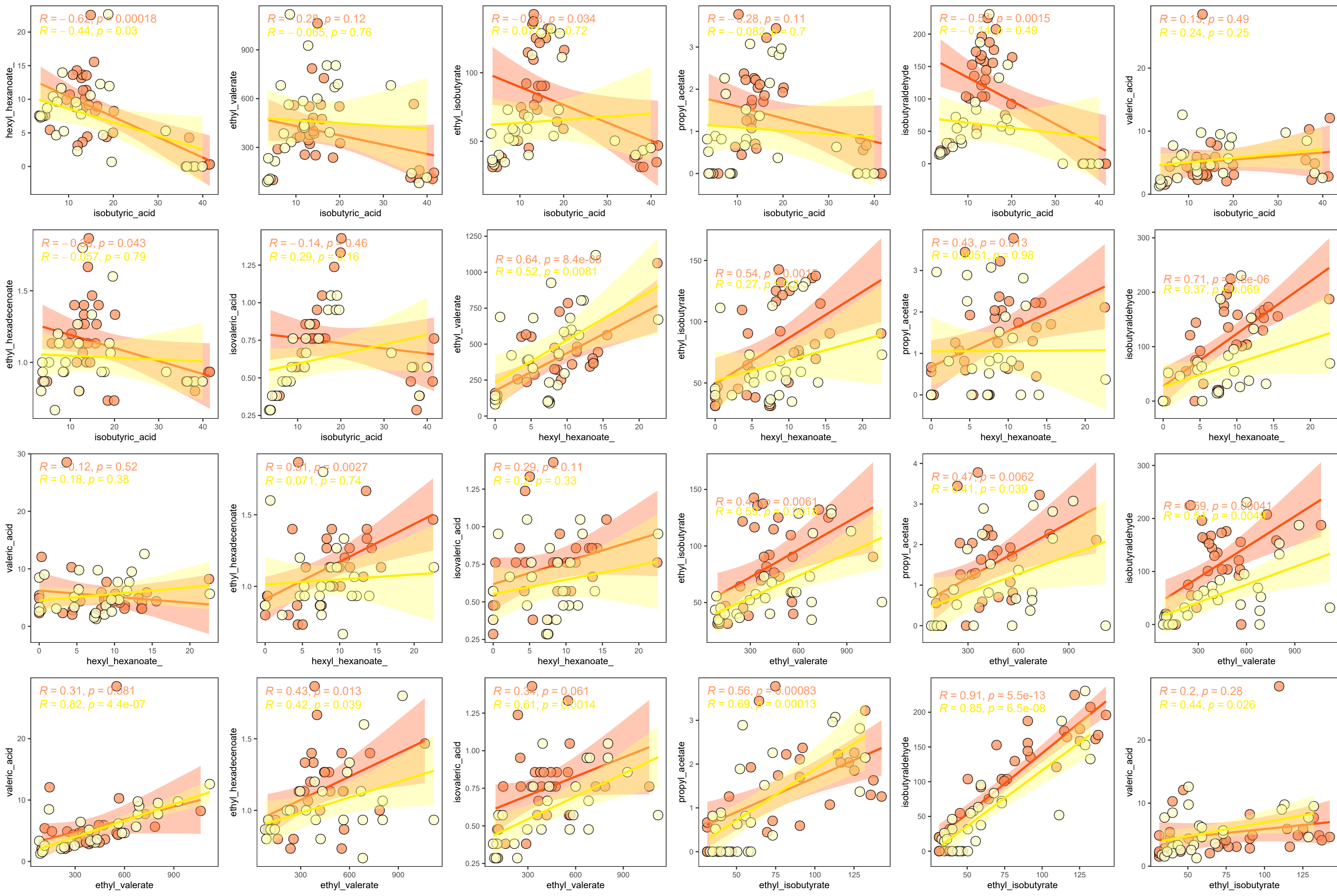

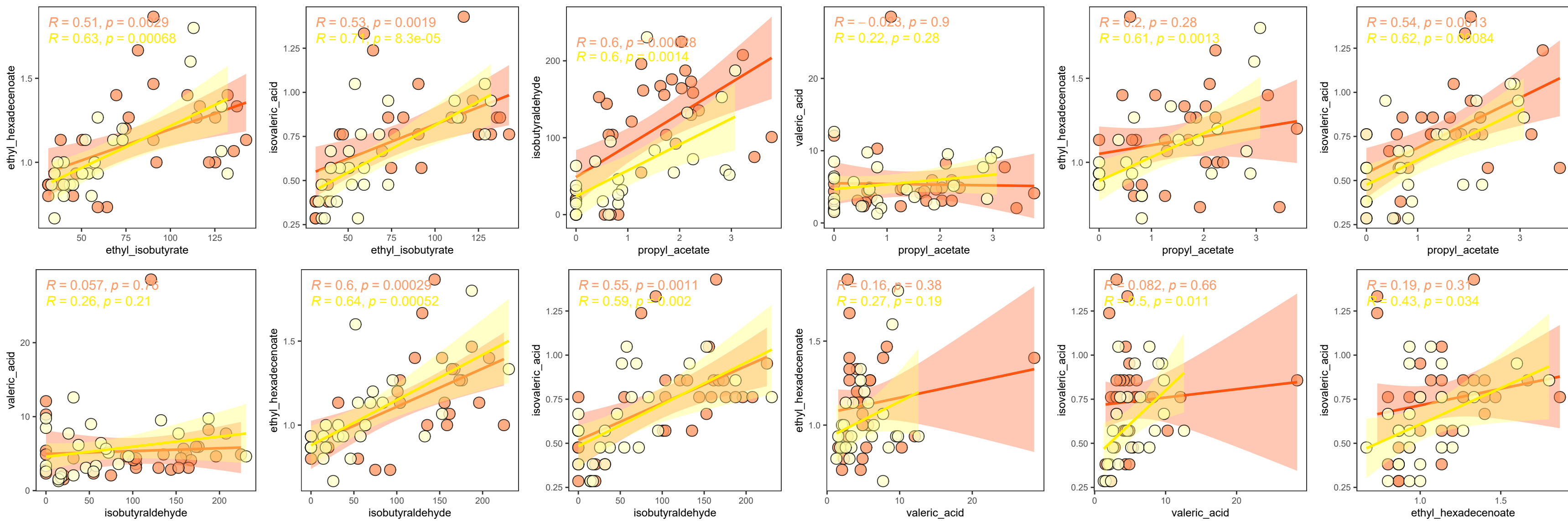

Supplement: Supplementary file 1 [file foods-11-03916-s001.zip › Supplementary File S1.pdf]
